# Preclinical toxicity and pharmacokinetics of a new orally bioavailable flubendazole formulation and the impact for clinical trials and risk/benefit to patients

**DOI:** 10.1371/journal.pntd.0007026

**Published:** 2019-01-16

**Authors:** Sophie Lachau-Durand, Lieve Lammens, Bas-jan van der Leede, Jacky Van Gompel, Graham Bailey, Marc Engelen, Ann Lampo

**Affiliations:** 1 Drug Metabolism and Pharmacokinetics, Janssen Research & Development, A Division of Janssen Pharmaceutica NV, Beerse, Belgium; 2 Nonclinical Safety, Janssen Research & Development, A Division of Janssen Pharmaceutica NV, Beerse, Belgium; 3 Project Management Office, Janssen Research & Development, A Division of Janssen Pharmaceutica NV, Beerse, Belgium; Swiss Tropical and Public Health Institute, SWITZERLAND

## Abstract

**Background:**

Flubendazole, originally developed to treat infections with intestinal nematodes, has been shown to be efficacious in animal models of filarial infections. For treatment of filarial nematodes, systemic exposure is needed. For this purpose, an orally bioavailable amorphous solid dispersion (ASD) formulation of flubendazole was developed. As this formulation results in improved systemic absorption, the pharmacokinetic and toxicological profile of the flubendazole ASD formulation have been assessed to ensure human safety before clinical trials could be initiated.

**Methods & findings:**

Safety pharmacology, toxicity and genotoxicity studies have been conducted with the flubendazole ASD formulation.

In animals, flubendazole has good oral bioavailability from an ASD formulation ranging from 15% in dogs, 27% in rats to more than 100% in jirds. In *in vivo* toxicity studies with the ASD formulation, high systemic exposure to flubendazole and its main metabolites was reached. Flubendazole, up to high peak plasma concentrations, does not induce C_max_ related effects in CNS or cardiovascular system. In repeated dose toxicity studies in rats and dogs, flubendazole-induced changes were observed in haematological, lymphoid and gastrointestinal systems and in testes. In dogs, the liver was an additional target organ. Upon treatment cessation, at least partial recovery was observed for these changes in dogs. In rats, the No Observed Adverse Effect Level (NOAEL) was 5 mg (as base)/kg body weight/day (mg eq./kg/day) in males and 2.5 mg eq./kg/day in females. In dogs, the NOAEL was lower than 20 mg eq./kg/day. Regarding genotoxicity, flubendazole was negative in the Ames test, but positive in the *in vivo* micronucleus test.

**Conclusions:**

Based on these results, in combination with previously described genotoxicity and reproductive toxicity data and the outcome of the preclinical efficacy studies, it was concluded that no flubendazole treatment regimen can be selected that would provide efficacy in humans at safe exposure.

## Author summary

This article describes pharmacokinetic profiles and results of safety pharmacology, toxicity and genotoxicity studies with an oral ASD formulation of flubendazole with improved bioavailability. Flubendazole administered as ASD formulation has good oral bioavailability in animals ranging from 15% to more than 100%. In *in vivo* toxicology studies, increased systemic exposure does not induce C_max_-related effects in CNS and cardiovascular systems. Increased exposure upon repeated dosing results in changes in haematological, lymphoid and gastrointestinal systems and in testes. In dogs, the liver was an additional target organ. These changes were at least partially reversible. Flubendazole is negative in the Ames test but positive in the *in vivo* micronucleus test. Because of the carcinogenic risk associated with this positive effect in the *in vivo* micronucleus test, exposure duration in patients should not exceed one day. Flubendazole-induced toxicity and associated risk is monitorable and controllable in patients if stringent precautions are applied in view of testicular toxicity and previously described teratogenicity. Considering both, treatment regimen needed for efficacy and outcome of toxicity and genotoxicity studies, it was concluded that the risk/benefit associated with the use of orally bioavailable flubendazole for the treatment of onchocerciasis in the field does not support further development.

## Introduction

Onchocerciasis is a neglected tropical disease caused by the parasitic worm species *Onchocerca volvulus*, which spreads through bites from infected black flies. The presence of larvae in the skin causes several symptoms, including intolerable itching. Larvae can migrate to the eye and cause decreased vision and blindness. Adult worms live in nodules in the skin, can survive for 10–15 years and produce thousands of larvae per day. Current treatment of onchocerciasis relies on three drugs, albendazole, ivermectine and diethylcarbamazine, agents working primarily against microfilarial stages. In tropical medicine, there is a need for a macrofilaricidal drug that safely kills adult filarial worms. [1, 2]

Flubendazole, originally discovered and developed by researcher Dr. Paul Janssen and his team, was first approved for human use in 1980 to treat soil transmitted helminths (STH), also known as intestinal worms. This methylcarbamate benzimidazole anthelmintic has been shown to have a marked macrofilaricidal effect on many filarial species. [2] Oral flubendazole formulations commercialized for the treatment of STH (trademarks Flubenol, Fluvermal and others) are very poorly absorbed. For treatment of STH, flubendazole acts locally in the gut. *In vivo* activity of flubendazole against a variety of filariid species has been reported in animals and man after parenteral administration indicating systemic exposure is needed for the treatment of onchocerciasis. For this purpose, an orally bioavailable amorphous solid dispersion (ASD) formulation was developed. ASD is an approach to formulate poorly water-soluble drugs in the amorphous form, for the enhancement of dissolution rate and bioavailability.

Because of the significantly higher systemic exposure anticipated from the ASD formulation, the safe evaluation of this formulation in clinical trials could not be supported by nonclinical safety studies performed for the marketed oral flubendazole formulation. In the nonclinical safety studies performed in support of flubendazole for STH, systemic exposure was very low. The set of nonclinical studies performed for the safety evaluation of the ASD formulation and their design including number of animals used, were based on international standards described in guidelines of the International Conference on Harmonisation (ICH) of Technical Requirements for Registration of Pharmaceuticals for Human Use. ICH guidelines promote safe and ethical development of pharmaceuticals and reducing the use of animals in accordance with the 3R (reduce/refine/replace) principles is part of their objectives.

With the ASD formulation, two-week repeated dose toxicity studies followed by 1-month recovery were performed in rats and dogs as well as a cardiovascular safety study in dogs and a CNS safety study in rats. Flubendazole amorphous solid dispersion had been tested in an *in vivo* micronucleus test [1] and an explorative oral embryofetal developmental toxicity study in the rat [3]. Flubendazole was also tested in an *in vivo* micronucleus test in rats as a solution/suspension in polyethylene glycol 400 (PEG400) and hydroxypropyl-β-cyclodextrin. An Ames test was performed with flubendazole. The results of the evaluation of flubendazole and its main metabolites in the Ames test and the *in vitro* micronucleus test have been described [1].

This paper describes the results of these preclinical safety studies and their impact/implications for the potential use of this new orally bioavailable ASD formulation in humans, for the conduct of clinical trials and finally for the risk/benefit associated with the use of orally bioavailable flubendazole for the treatment of onchocerciasis in the field.

## Results

### Pharmacokinetics

After intravenous administration, flubendazole (FBZ) showed a low plasma clearance in rodents (rats and jirds) and high clearance in dogs (Table 1), a medium volume of distribution and a short half-life in the 3 species. After oral administration, the bioavailability is > 100% in jirds, 27% in rats and 15% in dogs assuming the same clearance after intravenous and oral administration. Two metabolites of flubendazole were measured in plasma: the hydrolyzed flubendazole (H-FBZ) and the reduced flubendazole (R-FBZ) as these 2 metabolites might be potentially active. (Table 2) The plasma exposure ratios between parent and H-FBZ were 0.5 in jirds, 0.9 in rats and 1.7 in dogs. The plasma exposure ratios between parent and R-FBZ were 0.04 in jirds, 0.35 in rats and 2.8 in dogs.

**Table 1:** Plasma pharmacokinetic parameters after intravenous and oral administration of ASD formulation of flubendazole (mean ± standard deviation).

		Jird	Rat	Dog
After intravenous administration (n = 3)	Dose (mg/kg) of FBZ	1	2	0.5
	C_0_ (ng/mL)	ND	2356 ± 199	505 ± 21^a^
	AUC_0-inf_ (ng.h/mL)	752 ± 176	3390 ± 480	246 ± 47
	CL (mL/min/kg)	21 ± 5	10 ± 0.09	31 ± 6
	t_1/2_ (h)	1.1 ± 0.2	2.8 ± 0.32	1.2 ± 0.1
	Vd_ss_ (L/kg)	2.0 ± 0.3	2.4 ± 0.63^b^	1.5 ± 0
After oral administration of ASD formulation (n = 3)	Dose (mg/kg) of FBZ	20	20	35
	C_max_ (ng/mL)	6540 ± 191	1130 ± 279	665 ± 80
	t_max_ (h)	0.5	0.67	0.5–2
	AUC_0-inf_ (ng.h/mL)	21643 ± 4747	9100 ± 2810	2414 ± 365^c^
	F (%)	>100	27	15

ND: not determined

C_0_: extrapolated concentrations at t_0_, C_max_: maximum concentration, t_max_: time to reach the C_max_

AUC_0-inf_: the area under the curve from time zero to infinity CL: clearance, t_1/2_: half-life, Vd_ss_: volume of distribution at steady state, Vd: volume of distribution, F: bioavailability: calculated using the exposures obtained after oral and intravenous administration normalized by the corresponding doses assuming the same clearance after intravenous and oral administration. The value higher than 100% was the result of variability due to different animals being dosed for oral and intravenous administration. In addition, the difference in dose for the oral and intravenous administration could also have played a role as the clearance could not be exactly the same with different doses.

^a^: at end of infusion

^b^: Vd

^C^: AUC_0-24h_

**Table 2:** Plasma pharmacokinetic parameters of H-FBZ and R-FBZ after oral administration of ASD formulation (mean ±standard deviation).

	Jird		Rat		Dog	
Dose (mg eq/kg) of FBZ	20		20		35	
	H-FBZ	R-FBZ	H-FBZ	R-FBZ	H-FBZ	R-FBZ
C_max_ (ng/mL)	1190 ± 128	161± 53	473 ± 126	321 ± 118	346 ± 48	2077 ± 346
t_max_ (h)	2	1	1.7	1.7	2	0.5–1
AUC_0-inf_ (ng.h/mL)	11500 ± 2338	895 ± 348	8240 ± 1950	3230 ± 1130	4231 ± 308^a^	7042 ± 334^a^

^a^: AUC_0-24h_

Data available from the Dryad digital repository: https://doi.org/10.5061/dryad.5vv774m [4]

### Safety pharmacology studies

#### Cardiovascular safety study in dogs

When compared to vehicle dosing, a single oral administration of flubendazole at a dose of 20, 50 or 120 mg eq./kg to telemetered male beagle dogs did not exert any adverse effect on health status, body weight, electrocardiogram, body temperature, arterial blood pressure and respiratory parameters. Concentrations of flubendazole and the two metabolites R-FBZ and H-FBZ, measured approximately 6.5 hours after dosing at 20, 50 or 120 mg eq./kg, indicated exposure to flubendazole and both metabolites in all five dogs (Table 3).

**Table 3:** Plasma concentrations (ng/mL; mean ± standard deviation) of flubendazole, H-FBZ and R-FBZ at 6.5 h after dosing of single dose of ASD formulation of flubendazole in dogs.

Dose (mg eq./kg)	No. of dogs	flubendazole	H-FBZ	R-FBZ
20	5	44.3 ± 21	171 ± 54	132 ± 72
50	5	135 ± 71	226 ± 65	431 ± 214
120	5	186 ± 103	278 ± 102	604 ± 328

#### CNS safety study in rats

Under the experimental conditions of the study, a single oral administration of flubendazole at a dose of 5, 15 or 60 mg eq./kg to male Sprague-Dawley rats did not adversely affect body weight, rectal temperature or neuro-behavioral parameters investigated in the modified Irwin test. The concentrations of flubendazole and the two metabolites were measured after completion of the modified Irwin test sessions, by treating the 6 rats previously treated with the vehicle with a dose of 60 mg eq./kg. Toxicokinetic parameters were calculated (Table 4).

**Table 4:** Plasma toxicokinetic parameters of flubendazole, H-FBZ and R-FBZ after oral administration of ASD formulation at 60 mg eq./kg in rats (6 male rats) (Irwin Study).

	C_max_ (ng/ml, mean ± s.d.)	t_max_ (h)	AUC_0-24h_ (ng.h/ml, mean)*
Flubendazole	4330 ± 490	2	31700
H-FBZ	1420 ± 20	7	24700
R-FBZ	1940 ±800	2	24900

s.d.: standard deviation

*: no s.d. reported

Data from safety pharmacology studies available from the Dryad digital repository: https://doi.org/10.5061/dryad.5vv774m [4]

### 2-week repeated dose toxicity studies

#### 2-week repeated dose toxicity study in rats

Flubendazole dosed orally to male and female rats for 14 or 15 consecutive days resulted in premature sacrifice of all high dose animals (males at 30 mg eq./kg/day and females at 10 mg eq./kg/day) on Days 7, 8 or 11, due to poor general condition. Flubendazole-related mucosal atrophy in the small and large intestines and bone marrow atrophy were considered to be the main contributors to the moribund condition of these rats.

The primary effects associated with flubendazole administration involved the haematological and gastrointestinal systems. Additional effects were seen in the testes and thymus.

At the low dose (5 mg eq./kg/day in males and 2.5 mg eq./kg/day in females), no flubendazole-related adverse effects were observed.

At the mid dose, males (15 mg eq./kg/day) exhibited a flubendazole-related decrease in body weight gain from Day 8 onwards, which correlated with decreased food consumption. (Tables 5 and 6) Slight to moderate effects on haematology parameters were noted in the mid dose males (15 mg eq./kg/day) and females (5 mg eq./kg/day). Haematological effects included, but were not limited to, slight to moderate decreases in white blood cells (neutrophils and monocytes) and slight to moderate increases in red blood cell distribution width in both sexes. In addition, an increase in fibrinogen was noted in males and decreases in red blood cells, haemoglobin and haematocrit were noted in females, when compared to vehicle control. (Table 7) Organ weights were slightly to markedly lower in males (15 mg eq./kg/day) and females (5 mg eq./kg/day) for thymus, testes, prostate and/or spleen. (Table 8) Macroscopically, flaccid testes and reduced size of thymus were noted (Table 9); the microscopic correlates were tubular and germ cell degeneration and lymphoid depletion, respectively. The testicular changes were associated with changes in the epididymides (luminal cell debris and reduced luminal sperm). Other microscopic findings were noted in the spleen (increased hemosiderin pigmentation in males), bone marrow (atrophy in both sexes), Peyer’s patches (lymphoid depletion in males and females) and mesenteric lymph nodes (reduced germinal centers and lymphoid depletion in males). (Tables 10 and 11)

**Table 5:** Body weight gain (% difference in body weight compared to body weight on Day 1, mean value ± standard deviation) in rats in 2-Week toxicity study with oral ASD formulation (excluding satellite animals).

Dose group	Vehicle	Low Dose	Mid Dose	High Dose
Males-Dose Levels	0 mg eq./kg/day	5 mg eq./kg/day	15 mg eq./kg/day	30 mg eq./kg/day
No. of animals	15	10	10	15
Day 7	+6 ± 4.6	+7 ± 2.0	+5 ± 2.6	-6 ± 10.2 **
Day 14	+16 ± 3.6	+14 ± 4.1	+11 ± 4.8 **	-
Females-Dose Levels	0 mg eq./kg/day	2.5 mg eq./kg/day	5 mg eq./kg/day	10 mg eq./kg/day
No. of animals	15	10	10	15
Day 7	+2 ± 4.1	+3 ± 2.9	+1 ± 3.1	-7 ± 7.2 **
Day 14	+6 ± 4.7	+6 ± 3.7	+3 ± 3.1	-

** Dunnett-test based on pooled variance significant at 1% level

**Table 6:** Food consumption (gram/animal/day, mean value ± standard deviation) in rats in 2-week toxicity study with oral ASD formulation (excluding satellite animals).

Dose group	Vehicle	Low Dose	Mid Dose	High Dose
Males-Dose Levels	0 mg eq./kg/day	5 mg eq./kg/day	15 mg eq./kg/day	30 mg eq./kg/day
No. of animals	15	10	10	15
Day 1–4	27 ± 2.0	27 ± 0.6	26 ± 0.6	18 ± 4.8 *
Day 4–8	28 ± 1.9	27 ± 0.2	25 ± 1.2	18 ± 0.8 **
Days 8–11	32 ± 1.8	30 ± 0.4	27* ± 0.7	-
Days 11–14	29 ± 2.0	27 ± 1.3	24* ± 0.6	-
Females-Dose Levels	0 mg eq./kg/day	2.5 mg eq./kg/day	5 mg eq./kg/day	10 mg eq./kg/day
No. of animals	15	10	10	15
Day 1–4	19 ± 1.5	18 ± 2.0	16 ± 1.0	13 ± 1.4 **
Day 4–8	19 ± 1.5	19 ± 0.8	17 ± 0.4	10 ± 3.9 *
Days 8–11	21 ± 1.4	21 ± 0.2	20 ± 0.9	-
Days 11–14	18 ± 0.6	18 ± 0.8	17 ± 0.4	-

*/** Dunnett-test based on pooled variance significant at 5% (*) or 1% (**) level

**Table 7:** Haematology in rats in 2-week toxicity study with oral ASD formulation: Parameters with flubendazole-induced changes (mean value ± standard deviation) (excluding satellite animals) on Day 15 (Vehicle, Low and Mid Dose Groups) or Day 7 or 8 (High Dose Groups).

Dose group	Vehicle	Low Dose	Mid Dose	High Dose^&^
Males-Dose Levels	0 mg eq./kg/day	5 mg eq./kg/day	15 mg eq./kg/day	30 mg eq./kg/day
No. of animals	15	10	10	15
White Blood Cells (WBC) 10E9/L	10.1 ± 3.0	7.4 ± 1.6 *	6.6 ± 1.9 **	4.3 ± 2.7
Neutrophils %WBC	17.0 ± 6.6	12.2 ± 4.++0	4.0 ± 2.2 ++	2.8 ± 2.8
Monocytes %WBC	2.1 ± 0.8	2.1 ± 0.5	1.0++ ± 0.7	0.3 ± 0.3
Red Blood Cells 10E12/L	8.00 ± 0.50	8.26 ± 0.31	7.42 ± 0.53 **	7.76 ± 1.25
Red Blood Cell Distribution Width %	12.8 ± 1.1	13.1 ± 0.9	15.3 ± 1.7 **	12.0 ± 0.9
Haemoglobin mmol/L	9.5 ± 0.5	9.8 ± 0.3	9.2 ± 0.4	9.2 ± 1.4
Haematocrit L/L	0.443 ± 0.021	0.458 ± 0.017	0.423 ± 0.019 *	0.415 ± 0.060
Fibrinogen g/L	2.76 ± 0.20	2.83 ± 0.28	4.34 ± 2.18 **	2.75 ± 0.98
Females-Dose Levels	0 mg eq./kg/day	2.5 mg eq./kg/day	5 mg eq./kg/day	10 mg eq./kg/day
No. of animals	15	10	10	15
White Blood Cells (WBC) 10E9/L	7.1 ± 2.0	6.6 ± 1.7	5.7 ± 1.1	3.3 ± 1.5
Neutrophils %WBC	10.6 ± 3.4	10.2 ± 4.8	4.6++ ± 1.7	1.4 ± 1.9
Monocytes %WBC	1.7 ± 0.7	1.6 ± 0.7	0.8++ ± 0.3	0.2 ± 0.3
Red Blood Cells 10E12/L	7.70 ± 0.34	7.54 ± 0.20	6.77 ± 0.52 **	6.68 ± 0.73
Red Blood Cell Distribution Width %	11.9 ± 0.6	12.5 ± 0.6	14.2 ± 0.8 **	11.4 ± 0.7
Haemoglobin mmol/L	9.3 ± 0.4	9.1 ± 0.3	8.3 ± 0.6 **	7.9 ± 0.9
Haematocrit L/L	0.421 ± 0.019	0.416 ± 0.013	0.374 ± 0.027 **	0.351 ± 0.037
Fibrinogen g/L	2.34 ± 0.20	2.34 ± 0.14	2.38 ± 0.22	3.35 ± 1.73

+/++ Steel-test significant at 5% (+) or 1% (++) level

*/** Dunnett-test based on pooled variance significant at 5% (*) or 1% (**) level; ^&^ high dose group not included in statistical analysis

**Table 8:** Organ weights (gram, mean value ± standard deviation) in rats in 2-week toxicity study with oral ASD formulation: (excluding satellite animals): Organs with flubendazole-induced weight changes.

Dose group	Vehicle	Low Dose	Mid Dose	High Dose
Males-Dose Levels	0 mg eq./kg/day	5 mg eq./kg/day	15 mg eq./kg/day	30 mg eq./kg/day
No. of animals	15	10	10	15
Thymus	0.403 ± 0.118	0.295 ± 0.077 *	0.159 ± 0.063 **	-
Spleen	0.730 ± 0.324	0.595 ± 0.074	0.564 ± 0.105	-
Testes	3.23 ± 0.27	3.17 ± 0.26	2.76 ± 0.52 *	-
Prostate	0.856 ± 0.173	0.707 ± 0.169	0.629 ± 0.120 **	-
Females-Dose Levels	0 mg eq./kg/day	2.5 mg eq./kg/day	5 mg eq./kg/day	10 mg eq./kg/day
No. of animals	15	10	10	15
Thymus	0.396 ± 0.118	0.301 ± 0.075 *	0.190 ± 0.070 **	-
Spleen	0.483 ± 0.051	0.433 ± 0.046	0.405 ± 0.056 **	-

*/** Dunnett-test based on pooled variance significant at 5% (*) or 1% (**) level

**Table 9:** Macroscopic findings in rats in 2-week toxicity study with oral ASD formulation on Day 15 (Vehicle, Low and Mid Dose Groups) or Day 7 or 8 (High Dose Groups): Incidence per dose group of flubendazole-induced changes (excluding satellite animals).

Dose group	Vehicle	Low Dose	Mid Dose	High Dose^&^
Males-Dose Levels	0 mg eq./kg/day	5 mg eq./kg/day	15 mg eq./kg/day	30 mg eq./kg/day
No. of animals	10	10	10	15
Testes: flaccid	0	0	5#	9
Thymus: reduced in size	0	0	6#	14
Females-Dose Levels	0 mg eq./kg/day	2.5 mg eq./kg/day	5 mg eq./kg/day	10 mg eq./kg/day
No. of animals	10	10	10	15
Thymus: reduced in size	0	0	4	13

# Fisher’s Exact test significant at 5% (#)

^&^ high dose group not included in statistical analysis

**Table 10:** Microscopic findings in male rats in 2-week toxicity study with oral ASD formulation on Day 15 (Vehicle, Low and Mid Dose Groups) or Day 7 or 8 (High Dose Groups): Incidence per dose group of flubendazole-induced changes (excluding satellite animals).

Dose group	Vehicle	Low Dose	Mid Dose	High Dose
Males-Dose Levels	0 mg eq./kg/day	5 mg eq./kg/day	15 mg eq./kg/day	30 mg eq./kg/day
No. of animals	10	10	10	15
Bone marrow, femur: myeloid atrophy	0	0	10	15
Bone marrow, sternum: myeloid atrophy	0	0	10	15
Epididymides: luminal cell debris	0	0	10	15
Epididymides: reduced luminal sperm	0	0	10	15
Mesenteric lymph node: lymphoid depletion	0	0	1	3
Mesenteric lymph node: reduced germinal centers	0	0	4	10
Peyer’s patches: lymphoid depletion	0	0	3	3
Spleen: hemosiderin pigmentation	0	0	4	3
Testes: multinucleated giant cells	0	0	7	12
Testes: germ cell degeneration	0	0	9	15
Thymus: lymphoid depletion	0	0	6	15

**Table 11:** Microscopic findings in female rats in 2-week toxicity study with oral ASD formulation on Day 15 (Vehicle, Low and Mid Dose Groups) or Day 7 or 8 (High Dose Groups): Incidence per dose group of flubendazole-induced changes (excluding satellite animals).

Dose group	Vehicle	Low Dose	Mid Dose	High Dose
Females-Dose Levels	0 mg eq./kg/day	2.5 mg eq./kg/day	5 mg eq./kg/day	10 mg eq./kg/day
No. of animals	10	10	10	15
Bone marrow, femur: myeloid atrophy	0	0	10	15
Bone marrow, sternum: myeloid atrophy	0	0	10	15
Mesenteric lymph node: reduced germinal centers	0	0	0	11
Peyer’s patches: lymphoid depletion	0	0	4	1
Thymus: lymphoid depletion	0	0	7	15

Based on the information above, the No Observed Adverse Effect Level (NOAEL) in this rat study was considered to be the low dose, i.e. 5 mg eq./kg/day for males and 2.5 mg eq./kg/day for females. The exposures were higher in female compared to male rats. The sex related difference for flubendazole is probably due to the differential expression of various sex-dependent forms of cytochrome P450s (CYP450) mediated by hormonal regulation, enzymes which are involved in the metabolism of flubendazole. In general, this gender-related difference in CYP450 expression and metabolism is more striking in the rat than in any other species, Plasma levels of flubendazole and both metabolites, R-FBZ and H-FBZ, were measured and toxicokinetic parameters at the end of dosing are shown in Table 12.

**Table 12:** Plasma toxicokinetic parameters of flubendazole, H-FBZ and R-FBZ at Day 14 in satellite rats in 2-week toxicity study with oral ASD formulation.

Dose (mg eq./kg/day) of flubendazole	No. of rats	PK Parameters	Flubendazole		H-FBZ		R-FBZ	
			M	F	M	F	M	F
Males: 5 Females: 2.5	6/sex	C_max_ (ng/mL) (mean ± s.d.)	1200 ± 260	1210 ± 250	413 ± 65	473 ± 164	291 ± 28	50.1 ± 6.4
		AUC_0-24h_ (ng.h/mL) (mean)*	5580	3740	5910	6810	1930	227^b^
Males: 15 Females: 5	6/sex	C_max_ (ng/mL) (mean ± s.d.)	1840 ± -	2000 ± 120	748 ± -	872 ± -	335 ± 32	89.8 ± 20.2
		AUC_0-24h_ (ng.h/mL) (mean)*	8500	10500	10100	14300	3100	505^b^
Males: 30 Females: 10	6/sex	C_max_ (ng/mL) (mean ± s.d.)	2340 ± 1080	2550 ± 390^a^	1150^a^ ± 170	1280^a^ ± 300	557 ± 73^a^	133^a^ ± 39
		AUC_0-24h_ (ng.h/mL) (mean)*	10300^a^	15200^a^	16700	21800^a^	3120^a^^,^^b^	1330^a^

M: male, F: female

^a^: Day 10

^b^: AUC_0-7h,_ -: not reported, s.d.: standard deviation

*: standard deviation not reported

#### 2-week repeated dose toxicity study in dogs followed by a 1-month recovery period

Flubendazole dosed orally to male and female dogs for 14 or 15 consecutive days resulted in the premature sacrifice of one dog at 100 mg eq./kg/day, based on poor general condition. Flubendazole-related liver enzyme activity increase and associated hepatocellular necrosis were considered the main cause of morbidity. The primary effects associated with flubendazole administration involved the haematological and gastrointestinal systems. Additional effects were seen in the liver, testes, epididymides, thymus, lymph nodes and bone marrow.

Gastrointestinal clinical signs (fecal changes, diarrhea, vomiting) were observed at 40 and 100 mg eq./kg/day. Dogs dosed at 20 or 40 mg eq./kg/day minimally lost weight during the dosing period (-2% to -3%) while a more pronounced body weight loss was noted in dogs dosed at 100 mg eq./kg/day (-9% in males and -19% in females). (Table 13) At all dose levels, liver enzyme activities were increased, when compared to vehicle control. At 100 mg eq./kg/day, this was associated with an increase in bilirubin. (Table 14) Decreases in white blood cells and reticulocytes were noted only at the high dose of 100 mg eq./kg/day. (Table 15) Microscopic findings were noted in the liver (centrilobular hepatocellular necrosis and perivascular inflammatory infiltrate), testes (degenerative changes), epididymides (luminal cell debris) and stomach (increased apoptosis; females only) starting at 20 mg eq./kg/day; thymus (increased severity lymphoid depletion), female lymph nodes (lymphoid depletion) and male bone marrow (increased adipocytes) starting at 40 mg eq./kg/day and stomach (atrophy of the surface epithelium, hypertrophy of single mucus cells, haemorrhage and vacuolation of parietal cells in some animals), intestines (mucosal atrophy and hemorrhage in some animals), male lymph nodes (lymphoid depletion), Peyer’s patches (lymphoid depletion) and female bone marrow (diffuse hypocellularity and increased adipocytes) at 100 mg eq./kg/day. (Tables 16 and 17)

**Table 13:** Body weight gain (% difference in body weight compared to body weight on Day 1, mean value ± standard deviation) in dogs in 2-week toxicity study with oral ASD formulation.

Dose group	Vehicle	Low Dose	Mid Dose	High Dose
Males-Dose Levels	0 mg eq./kg/day	20 mg eq./kg/day	40 mg eq./kg/day	100 mg eq./kg/day
No. of animals	5	3	5	5
End of treatment	+2 ± 1.5	-3 ± 2.7	-2 ± 1.5	-9 ± 3.6
Females-Dose Levels	0 mg eq./kg/day	20 mg eq./kg/day	40 mg eq./kg/day	100 mg eq./kg/day
No. of animals	5	3	5	5
End of treatment	+1 ± 1.9	-2 ± 3.5	-2 ± 3.4	-19 ± 3.7

**Table 14:** Clinical chemistry in dogs in 2-week toxicity study with oral ASD formulation: parameters with flubendazole-induced changes (mean value ± standard deviation) end of treatment.

Dose group	Vehicle	Low Dose	Mid Dose	High Dose
Males-Dose Levels	0 mg eq./kg/day	20 mg eq./kg/day	40 mg eq./kg/day	100 mg eq./kg/day
No. of animals	5	3	5	5
Alanine aminotransferase U/L	34.3 ± 9.3	114.5 ± 141.6	199.0 ± 160.3	46.7 ± 8.8
Aspartate aminotransferase U/L	30.0 ± 3.8	55.2 ± 39.0	65.4 ± 38.2	30.0 ± 4.8
Alkaline phosphatase U/L	93 ± 40	88 ± 42	111 ± 41	88 ± 20
Gamma glutamyl transferase U/L	3.3 ± 0.8	2.4 ± 0.5	3.7 ± 1.2	3.1 ± 0.5
Glutamate dehydrogenase U/L	3.1 ± 0.9	15.8 ± 21.1	17.9 ± 12.2	7.3 ± 1.5
Total bilirubin μmol/L	3.0 ± 0.4	3.1 ± 1.0	3.6 ± 0.7	3.5 ± 0.6
Females-Dose Levels	0 mg eq./kg/day	20 mg eq./kg/day	40 mg eq./kg/day	100 mg eq./kg/day
No. of animals	5	3	5	5
Alanine aminotransferase U/L	25.7 ± 7.3	169.9 ± 201.6	137.1 ± 94.6	296.2 ± 322.2
Aspartate aminotransferase U/L	28.4 ± 3.7	77.4 ± 59.4	58.2 ± 40.1	72.0 ± 13.3
Alkaline phosphatase U/L	74 ± 26	112 ± 11	146 ± 46	257 ± 318
Gamma glutamyl transferase U/L	2.9 ± 0.8	3.6 ± 1.1	4.7 ± 1.5	10.4 ± 9.7
Glutamate dehydrogenase U/L	4.7 ± 0.7	27.4 ± 25.5	23.5 ± 16.0	37.1 ± 48.1
Total bilirubin μmol/L	3.5 ± 0.3	4.6 ± 0.8	3.8 ± 0.4	17.9 ± 28.1

**Table 15:** Haematology in dogs in 2-week toxicity study with oral ASD formulation: parameters with flubendazole-induced changes (mean value ± standard deviation) end of treatment.

Dose group	Vehicle	Low Dose	Mid Dose	High Dose
Males-Dose Levels	0 mg eq./kg/day	20 mg eq./kg/day	40 mg eq./kg/day	100 mg eq./kg/day
No. of animals	5	3	5	5
White blood cells 10E9/L	9.8 ± 2.3	8.4 ± 4.2	7.4 ± 1.8	4.0 ± 1.3
Reticulocytes % red blood cells	0.9 ± 0.4	0.3 ± 0.2	0.5 ± 0.2	0.2 ± 0.1
Females-Dose Levels	0 mg eq./kg/day	20 mg eq./kg/day	40 mg eq./kg/day	100 mg eq./kg/day
No. of animals	5	3	5	5
White blood cells 10E9/L	8.6 ± 1.7	9.1 ± 1.3	7.6 ± 2.4	3.2 ± 0.7
Reticulocytes % red blood cells	0.6 ± 0.2	0.4 ± 0.2	0.4 ± 0.1	0.1 ± 0.0

**Table 16:** Microscopic findings in male dogs in 2-week toxicity study with oral ASD formulation end of treatment: incidence per dose group of flubendazole-induced changes.

Dose group	Vehicle	Low Dose	Mid Dose	High Dose
Males-Dose Levels	0 mg eq./kg/day	20 mg eq./kg/day	40 mg eq./kg/day	100 mg eq./kg/day
No. of animals	3	3	3	3
Bone marrow, femur: hypocellularity	0	0	0	1
Bone marrow, sternum: hypocellularity	0	0	0	0
Bone marrow, sternum: increased adipocytes	0	1	1	2
Cecum: mucosal atrophy	0	0	0	1
Colon: mucosal atrophy	0	0	0	1
Duodenum: mucosal atrophy	0	0	0	1
Epididymides: luminal cell debris	0	1	0	3
Ileum: mucosal atrophy	0	0	0	1
Jejunum: mucosal atrophy	0	0	0	1
Jejunum: haemorrhage	0	0	0	1
Liver: hepatocellular necrosis	0	1	2	1
Liver: perivascular inflammatory infiltrate	0	1	3	3
Mesenteric lymph node: lymphoid depletion	0	0	0	2
Peyer’s patches: lymphoid depletion	0	0	0	2
Stomach, pyloric region: increased apoptosis	0	0	0	2
Stomach, pyloric region: atrophy surface epithelium	0	0	0	1
Stomach, pyloric region: hypertrophy mucus cells	0	0	0	2
Stomach, pyloric region: haemorrhage	0	0	0	1
Testes: degeneration/depletion germ cells	0	1	1	3
Thymus: lymphoid depletion	1	1	1	2

**Table 17:** Microscopic findings in female dogs in 2-week toxicity study with oral ASD formulation end of treatment: Incidence per dose group of flubendazole-induced changes.

Dose group	Vehicle	Low Dose	Mid Dose	High Dose
Females-Dose Levels	0 mg eq./kg/day	20 mg eq./kg/day	40 mg eq./kg/day	100 mg eq./kg/day
No. of animals	3	3	3	3
Bone marrow, femur: hypocellularity	0	0	0	2
Bone marrow, sternum: hypocellularity	0	0	0	3
Bone marrow, sternum: increased adipocytes	1	0	0	2
Cecum: mucosal atrophy	0	0	0	2
Colon: mucosal atrophy	0	0	0	1
Duodenum: mucosal atrophy	0	0	0	2
Duodenum: haemorrhage	0	0	0	1
Ileum: mucosal atrophy	0	0	0	2
Jejunum: mucosal atrophy	0	0	0	2
Liver: hepatocellular necrosis	0	1	1	3
Liver: perivascular inflammatory infiltrate	0	3	3	2
Mandibular lymph node: lymphoid depletion	0	0	1	3
Mesenteric lymph node: lymphoid depletion	0	0	0	2
Peyer’s patches: lymphoid depletion	0	0	0	2
Stomach, fundic region: atrophy surface epithelium	0	0	0	1
Stomach, fundic region: vacuolation parietal cells	0	0	0	1
Stomach, pyloric region: increased apoptosis	0	2	1	1
Stomach, pyloric region: atrophy surface epithelium	0	0	0	1
Stomach, pyloric region: hypertrophy mucus cells	0	0	0	1
Stomach, pyloric region: haemorrhage	0	0	0	1
Thymus: lymphoid depletion	0	0	3	3

There was complete recovery for clinical signs, body weights, food consumption and haematology parameters at 40 and 100 mg eq./kg/day, and liver enzyme activities and microscopic findings at 40 mg eq./kg/day. There was partial recovery for alanine aminotransferase activity and spleen weight in females at 100 mg eq./kg/day and microscopic findings in the testes/epididymides and the perivascular inflammatory infiltrate in the liver at 100 mg eq./kg/day. Based on the information above, a NOAEL for dogs after 14 days of treatment could not be determined.

Plasma levels of flubendazole and both metabolites, reduced flubendazole and hydrolyzed flubendazole were measured and plasma toxicokinetic is shown in Table 18.

**Table 18:** Plasma toxicokinetic parameters of flubendazole, H-FBZ and R-FBZ in dogs at Day 14 in 2-week toxicity study with oral ASD formulation.

Dose (mg eq./kg) of flubendazole	No. of dogs	PK Parameters	flubendazole		H-FBZ		R-FBZ	
			M	F	M	F	M	F
20	3 dogs/sex	C_max_ (ng/mL) (mean ± s.d.)	1190 ± 500	1430 ± 740	478 ± 202	525 ± 99	2990 ± 960	2410 ± 400
		AUC_0-24h_ (ng.h/mL) (mean ± s.d.)	2610 ± 740	3050 ± 850	5160 ± 2260	6700 ± 580	8040 ± 2730	6990 ± 1190
40	3 dogs/sex	C_max_ (ng/mL) (mean ± s.d.)	1330 ± 430	1070 ± 310	557 ± 84	558 ± 120	4000 ± 1560	3330 ± 1020
		AUC_0-24h_ (ng.h/mL) (mean ± s.d.)	3420 ± 860	2950 ± 790	6810 ± 1090	6730 ± 950	10600 ± 3100	8810 ± 2640
100^a^	3 dogs/sex	C_max_ (ng/mL) (mean ± s.d.)	1610 ± 450	1850 ± 540	548 ± 87	694 ± 193	4570 ± 650	5500 ± 1970
		AUC_0-24h_ (ng.h/mL) (mean ± s.d.)	7150 ± 1990	12200 ± 4000	6830 ± 810	9680 ± 2950	27600 ± 7900	49200 ± 17500

^a^: 50 mg/kg b.i.d, s.d.: standard deviation

Data of 2-week repeated dose toxicity studies available from the Dryad digital repository: https://doi.org/10.5061/dryad.5vv774m [4]

### Genotoxicity assessments

#### Ames test

No biological relevant and/or dose-related increases in the number of revertant colonies were observed with any of the tester strains up to the precipitating concentration of 2000 μg/plate either in the presence or absence of an exogenous metabolic activation system (S9-mix). These results indicate that flubendazole was negative for the ability to induce reverse mutations at selected loci of several strains of Salmonella typhimurium and at the tryptophan locus of Escherichia coli strain WP2 uvrA in the presence and absence of S9 mix.

#### *In vivo* micronucleus test in rats

(not performed with ASD formulation but with aqueous solution/suspension in demineralized water containing 10% polyethylene glycol 400, 20% hydroxypropyl-β-cyclodextrin and HCl to pH 1.5± 0.1) In the peripheral blood micronucleus test, a slight reduction in red blood cell proliferation was observed in male rats dosed with flubendazole at 15 and 50 mg/kg/day. In female rats, a pronounced reduction in red blood cell proliferation was observed at 15 and 50 mg/kg/day. Administration of flubendazole did not result in a biologically relevant increase in micronucleated reticulocytes in male or female rats at any of the dose levels tested. (Table 19) In the bone marrow micronucleus test, a dose-related reduction in red blood cell proliferation was observed in female rats dosed with flubendazole at 5 mg/kg/day and above. Administration of flubendazole resulted in biologically relevant increases in micronucleated polychromatic erythrocytes in female rats at 5 mg/kg/day and above and in male rats at 50 mg/kg/day. (Table 20) Plasma analysis confirmed exposure of the rats in this study to flubendazole and its 2 metabolites, reduced flubendazole and hydrolyzed flubendazole. Toxicokinetic parameters are shown in Table 21.

**Table 19:** % reticulocytes (RETs) and % micronucleated reticulocytes (MN-RETs) in peripheral blood in *in vivo* micronucleus study in rats.

Males—Dose levels	Number of animals	% RETs	% MN-RETs
0 mg/kg/day+	5	2.83 ± 0.31 @@	0.08 ± 0.03 ~
0.15 mg/kg/day	5	2.88 ± 1.00	0.16 ± 0.05
0.5 mg/kg/day	5	2.80 ± 0.46	0.14 ± 0.04
1.5 mg/kg/day	5	3.01 ± 1.06	0.16 ± 0.06 &
5 mg/kg/day	5	2.44 ± 0.51	0.12 ± 0.05
15 mg/kg/day	5	2.00 ± 0.36 *	0.17 ± 0.04 &
50 mg/kg/day	5	1.70 ± 0.84	0.17 ± 0.05 &
150 mg/kg/day	5	2.38 ± 0.38	0.15 ± 0.07
10 mg/kg/day++	5	1.22 ± 0.24 $ $ $	1.26 ± 0.51 ###
Females—Dose levels	Number of animals	% RETs	% MN-RETs
0 mg/kg/day+	5	1.38 ± 0.38 @@@	0.10 ± 0.03 ~~
0.15 mg/kg/day	5	1.77 ± 0.36	0.12 ± 0.02
0.5 mg/kg/day	5	1.44 ± 0.53	0.11 ± 0.07
1.5 mg/kg/day	5	1.57 ± 0.45	0.11 ± 0.03
5 mg/kg/day	5	0.92 ± 0.28	0.16 ± 0.05
15 mg/kg/day	5	0.48 ± 0.27 **	0.16 ± 0.07
50 mg/kg/day	5	0.38 ± 0.34 **	0.15 ± 0.07^a^
150 mg/kg/day	5	0.91 ± 0.46	0.17 ± 0.06
10 mg/kg/day++	5	0.65 ± 0.22 $ $	0.85 ± 0.40 ##

Significance versus vehicle group by one-sided t-test: ## p<0.01, ### p<0.001

Significance versus vehicle by two-sided t-test: $ $ p<0.01, $ $ $ p<0.001

Significance versus vehicle group by one-sided Dunnett's test: & p<0.05

Significance versus vehicle group by two-sided Dunn's test: * p<0.05, ** p<0.01

Significance by one-sided Jonckheere-Terpstra Trend test: ~ p<0.05, ~~ p<0.01

Significance by two-sided Jonckheere-Terpstra Trend test: @@ p<0.01, @@@ p<0.001

+ Vehicle group

++ Positive control: cyclophosphamide

^a^ 3 animals

**Table 20:** % polychromatic erythrocytes (PCEs) and % micronucleated polychromatic erythrocytes (MN-PCEs) in bone marrow in *in vivo* micronucleus study in rats.

Males—Dose levels	Number of animals	% PCEs	% MN-PCEs
0 mg/kg/day+	5	52.98 ± 6.09	0.18 ± 0.12
0.15 mg/kg/day	5	53.82 ± 4.90	0.18 ± 0.09
0.5 mg/kg/day	5	57.78 ± 4.06	0.27 ± 0.13
1.5 mg/kg/day	5	52.20 ± 9.13	0.27 ± 0.11
5 mg/kg/day	5	54.44 ± 5.53	0.27 ± 0.08
15 mg/kg/day	5	50.94 ± 6.32	0.33 ± 0.10
50 mg/kg/day	5	47.54 ± 6.94	0.44 ± 0.27
150 mg/kg/day	5	47.26 ± 5.86	0.28 ± 0.14
10 mg/kg/day++	5	45.28 ± 4.77	3.46 ± 0.56 **
Females—Dose levels	Number of animals	% PCEs	% MN-PCEs
0 mg/kg/day+	5	48.98 ± 9.36	0.22 ± 0.08
0.15 mg/kg/day	5	55.00 ± 5.87	0.27 ± 0.08
0.5 mg/kg/day	5	49.60 ± 12.53	0.33 ± 0.22
1.5 mg/kg/day	5	49.34 ± 11.85	0.16 ± 0.10
5 mg/kg/day	5	36.85 ± 9.21	0.61 ± 0.46
15 mg/kg/day	5	25.96 ± 4.09 **	0.83 ± 0.42 **
50 mg/kg/day	5	24.08 ± 9.29 **	1.71 ± 0.74 **
150 mg/kg/day	5	40.64 ± 14.16	0.37 ± 0.21
10 mg/kg/day++	5	29.54 ± 4.18 **	2.32 ± 0.65 **

Significance versus vehicle group by two-sided Mann-Whitney U test: ** p<0.01

+ Vehicle group

++ Positive control: cyclophosphamide

**Table 21:** Plasma toxicokinetic parameters of flubendazole, H-FBZ and R-FBZ after 2 days of repeated oral dosing of a solution/suspension of flubendazole in demineralised water containing 10% polyethylene glycol 400 and 20% hydroxypropyl-β-cyclodextrin in rats in micronucleus study.

Dose (mg/kg) of flu bendazole	No. of rats	PK Parameters	flubendazole		H-FBZ		R-FBZ	
			M	F	M	F	M	F
0.15	3/sex	C_max_ (ng/mL) (mean ± s.d.)	11.7^a^ ± 2.60	88.9 ± 97.7^a^	15.1 ± 2.37	38.6 ± 23.2	4.14^c^	NC
		AUC_0-24h_ (ng.h/mL) (mean ± s.d.)	36.0^a^^,^^b^ ± 1.24	138^a^^,^ ^b^ ± 143	81.5^b^ ± 12.0	123^b^ ± 13.9	NC	NC
0.5	3/sex	C_max_ (ng/mL) (mean ± s.d.)	98.5 ±30.5	139 ± 25.9	42.3 ± 6.59	62.6 ± 5.68	31.7 ± 8.23	11.1 ± 2.58
		AUC_0-24h_ (ng.h/mL) (mean ± s.d.)	247^b^ ± 82.7	523^a^ ± 19.6	533^a^ ± 104	766 ± 43.8	116^b^ ± 20.6	54^b^ ± 17.8
1.5	3/sex	C_max_ (ng/mL) (mean ± s.d.)	311^a^ ± 37.5	547 ± 86.9	83.4 ± 70.3	273 ± 33.2	103^a^ ± 4.24	37.6 ± 9.76
		AUC_0-24h_ (ng.h/mL) (mean ± s.d.)	865^a^^,^^b^ ± 144	1539 ± 652	1438^a^ ± 415	2599 ± 85.7	401^b^ ± 33.1	173^a^ ± 120
5	3/sex	C_max_ (ng/mL) (mean ± s.d.)	787 ± 80.3	1393 ± 246	240 ± 76.3	535 ± 64.0	352 ± 83.1	158 ± 11.9
		AUC_0-24h_ (ng.h/mL) (mean ± s.d.)	3099 ± 529^a^	6955 ± 3076	3087 ± 369	8335 ± 2588	2022 ± 343^a^	1110 ± 262
15	3/sex	C_max_ (ng/mL)	1703 ± 749	2020 ± 972	348 ± 51.5	687 ± 226	548 ± 143	183 ± 42.8
		AUC_0-24h_ (ng.h/mL) (mean ± s.d.)	5018 ± 2594^a^	10242 ± 5383	4241 ± 622	13435 ± 6727	2852^a^ ± 1039	1880^a^ ± 281
50	3/sex	C_max_ (ng/mL) (mean ± s.d.)	1141 ± 398	2413 ± 946	511 ± 293	1308 ± 811	548 ± 218	363 ± 131
		AUC_0-24h_ (ng.h/mL) (mean ± s.d.)	6001 ^a^ ± 2788	17046 ± 10370	6263 ± 1565	28049 ± 31613	4433^a^ ± 1707	3925 ± 2131
150	3/sex	C_max_ (ng/mL) (mean ± s.d.)	866 ± 97.5	1290 ± 494	412 ± 163	628 ± 209	310 ± 58.8	108 ± 30.1
		AUC_0-24h_ (ng.h/mL) (mean ± s.d.)	4644 ± 1722	7603 ± 1799	6204 ± 3579	10430 ± 5923	3166^a^ ± 1347	1158^a^ ± 283

^a^: n = 2

^b^: AUC_0-7h_

^c^: n = 1

Data of genotoxicity assessments available from the Dryad digital repository: https://doi.org/10.5061/dryad.5vv774m [4]

Based on the results of this study, it is concluded that flubendazole induces structural and/or numerical chromosome aberrations in erythrocytes of rat bone marrow. There were no biologically relevant increases in micronuclei in male rats up to the 15 mg/kg/day dose, however, the lowest dose to cause biologically relevant induction of micronuclei in female rats was 5 mg/kg/day.

## Discussion

Flubendazole as currently formulated for the treatment of gastrointestinal parasites has poor systemic availability when given orally. Due to its poor bioavailability and solubility, the original oral formulation induces limited systemic or gastrointestinal toxicities and is negative in the bone marrow micronucleus tests. [1] Flubendazole has been shown to be highly efficacious in animal models of filarial infections when dosed subcutaneously. The clinical efficacy of flubendazole for treatment of onchocerciasis has also been reported after intramuscular administration of a suspension in humans. However, intramuscular administration resulted in serious injection site reactions. [5] Consequently, it is preferred to increase systemic availability of flubendazole by improved oral formulations. [6] Several orally bio-available formulations were developed. The oral ASD formulation was selected for preclinical development. As the new oral ASD formulation resulted in improved systemic absorption, preclinical development with this formulation did not only include extensive evaluation of efficacy and pharmacokinetics but also a re-evaluation of the toxicity of flubendazole including potential genotoxicity. It was anticipated that increased systemic exposure to flubendazole would result in toxicity related to the tubulin binding, the mechanism of action of flubendazole. Therefore, the purpose of the additional efficacy, pharmacokinetic and toxicity studies with the ASD formulation was to evaluate whether a treatment regimen could be identified that would result in an acceptable risk/benefit profile.

*In vivo* oral toxicity studies with the ASD formulation described above, clearly show relevant systemic exposure to flubendazole and its main metabolites, reduced flubendazole and hydrolyzed flubendazole. (Tables 3, 4, 12 and 18) In the rat and dog, the highest exposures reached at the end of 2-week repeated dosing (data in Tables 12 and 18) were multiple times higher when compared with systemic exposure in humans (less than 1 ng/ml C_max_ in plasma) following treatment with the marketed flubendazole formulation at the usual dosage (100 mg once or twice a day for 3 days). [1] In the preclinical *in vivo* filarial efficacy studies described by MP Hübner et al., 100% efficacy could not be achieved with oral treatment. Highest efficacy achieved with oral treatment with the ASD formulation was 90% in the *Litomosoides* jird model at 15 mg eq./kg/day for 10 days [7]. One hundred % efficacy was only observed with subcutaneous treatment in several models of infection at 10 mg/kg/day for 5 days. This subcutaneous treatment was associated with a C_max_ of 50 ng/ml and an AUC_0-24h_ of 1100 ng.h/ml, both on day 5 (data of Litomosoides sigmodontis study in female jirds [7]). Notwithstanding lower efficacy, oral treatment at 15 mg eq./kg/day for 10 days was associated with much higher exposures. On day 10, C_max_ was 3300 ng/ml and AUC_0-24h_ was 20000 ng.h/ml (data of Litomosoides sigmodontis study in female jirds [7]). The pharmacokinetic profile after a single subcutaneous and a single oral dose was very different. After an oral dose at 15 mg eq./kg, the plasma concentration decreased very rapidly, dropping below the limit of quantification after 72 hours while after a single subcutaneous injection at 10 mg/kg, sustained plasma levels were observed for at least 3 months [7]. These data indicate that prolonged exposure at low plasma concentration is probably more important for efficacy than reaching high plasma concentrations. Comparing exposures required for efficacy and those achieved in toxicity studies, we can conclude that the exposure in the oral toxicity studies with the ASD formulation, did not fully cover the exposure after oral treatment at 15 mg eq./kg/day for 10 days in the *Litomosoides* jird model which was associated with 90% efficacy. However, when comparing with C_max_ and AUC _0-24h_ of the subcutaneous treatment regimen associated with 100% efficacy in the *Litomosoides* jird model, exposures in the oral toxicity studies with the ASD formulation were higher.

In the CNS safety study in rats and the cardiovascular safety study in dogs, a single dose up to 60 mg eq./kg in the rat and 120 mg eq./kg in the dog, did not result in relevant CNS findings in the rat or cardiovascular findings in the dog. In the rat, the dose of 60 mg eq./day was associated with a peak exposure of 4330 ng/ml. (Table 4) In the dog, peak exposure was not determined but plasma concentrations measured 6.5 hours after dosing demonstrated exposure to flubendazole and its main metabolites. Mean plasma concentration of flubendazole at 120 mg eq./day 6.5 hours after dosing was 186 ng/ml. (Table 3) This is however an underestimation of the peak exposure since C_max_ at 50 mg eq./kg/day b.i.d. in the 2-week repeated dose toxicity study in male dogs on day 1 was 1660 ng/ml. These data indicate that flubendazole up to high peak plasma concentrations, does not induce C_max_ related effects in the central nervous system or cardiovascular system.

In the 2-week repeated dose toxicity studies in rats and dogs, main flubendazole-related changes were observed in haematological and lymphoid systems, gastrointestinal system and testes. In the dog, the liver was an additional target organ of toxicity of flubendazole. (rat data in Tables 7, 8, 9, 10 and 11; dog data in Tables 14, 15, 16 and 17) Flubendazole-induced changes in haematological, lymphoid and gastrointestinal systems were considered the consequence of its binding to tubulin. Like many benzimidazoles, flubendazole binds to the tubulin protein to the same site as colchicine. This binding results in inhibition of the polymerization of tubulin and thus disruption of the mitotic spindle. [1] For colchicine it is described that disruption of the microtubular network results in arrest of mitosis in metaphase because chromosome separation depends on microtubular function, thus inhibiting cell division. The organ systems with the highest turnover rates such as bone marrow and the gastrointestinal tract are the most vulnerable and most readily affected. [8] These organ systems have also been described to be affected by treatment with other benzimidazoles which is in line with their similar mechanism of action (tubulin binding). [9, 10] Dosing with other benzimidazoles like albendazole, mebendazole and oxfendazole in preclinical toxicity studies also resulted in testicular changes. [9, 10] For oxfendazole, the mechanisms underlying the testicular toxicity are most probably disruption of the microtubules, and degeneration of the Sertoli cells. [11] Regarding the liver toxicity observed in dogs treated with the ASD-formulation of flubendazole, there is no obvious link with the binding to tubulin by flubendazole.

These flubendazole-induced changes were at least partially reversible as shown in the dog 2-week toxicity study after 1-month recovery.

From a quantitative point of view, flubendazole-related changes were observed in the rat 2-week toxicity study at the mid dose and above with the low dose, i.e. 5 mg eq./kg/day for males and 2.5 mg eq./kg/day for females being the NOAEL (exposures associated with NOAEL in Table 12). In the dog 2-week toxicity study, flubendazole-related changes were observed at the low dose and the NOAEL could not be established. Since we do not know the NOAEL in the dog study, in case of clinical trials, careful evaluation for potential flubendazole-induced changes would be warranted by monitoring for gastrointestinal symptoms, haematology and clinical chemistry changes for potential effects on bone marrow and liver and by sperm evaluation for potential testicular toxicity. Contraception for males during 3 months after dosing should be recommended because of the risk of potential impact of testicular toxicity on offspring. Since the testicular toxicity is probably, at least partially, related to disruption of the microtubular network and hence potential disruption of chromosome separation, it can be associated with chromosomal damage. For DNA damage, it has been described that it can be transmitted from fathers to offspring [12].

Results of an explorative oral embryofetal developmental toxicity study in the rat have been published by M. Longo et al. [2] They also used a flubendazole-ASD formulation (developed and provided by Abbvie) at dose levels of flubendazole of 2, 3.46 and 6.32 mg eq./kg/day. Pregnant female Sprague-Dawley rats were dosed on gestational day (GD) 9.5 and 10.5 and embryos/fetuses were evaluated on GD 11.5, 12.5 or 20. At 2 mg eq./kg/day, flubendazole did not interfere with rat embryofetal development (C_max_ = 389 ng/ml, AUC_0-24h_ = 2190 ng.h/ml after single administration). From 3.46 mg eq./kg/day (C_max_ = 546 ng/ml, AUC_0-24h_ = 4830 ng.h/ml after single administration) onward, flubendazole markedly reduced embryonic development by GD 12.5. On GD 20, 80% of fetuses showed malformations. [2] Based on these results, flubendazole is considered a strong teratogen. Inclusion of women of childbearing potential should therefore, only be considered in well controlled clinical trials of limited size with appropriate contraceptive measures.

Flubendazole is a potent aneugen *in vitro*. This aneugenicity is the consequence of the binding of flubendazole to the tubulin protein, inhibiting polymerization of tubulin and consequently causing mitotic spindle disruption. Spindle poisons all have the potential to induce polyploidy and aneugenicity. [1] The hydrolyzed metabolite of flubendazole is negative in the *in vitro* MNT, but the reduced metabolite (R- and S-forms) shows both aneugenic and clastogenic activity. Like flubendazole itself, both metabolites are negative in the Ames test. [1] The *in vivo* micronucleus test with the ASD formulation of flubendazole published by Tweats et al. also showed evidence of induced aneugenicity. [1] This is in line with the results of the *in vivo* micronucleus test with an aqueous solution/suspension of flubendazole in demineralised water containing polyethylene glycol 400, hydroxypropyl-β-cyclodextrin and HCl, described in this article. In this study, flubendazole induced structural and/or numerical chromosome aberrations in erythrocytes of rat bone marrow from a dose of 5 mg/kg/day onwards, at similar exposure levels as with the ASD formulation. (Table 20) [1] Aneugens are accepted as having threshold dose responses with a clear mode of action, which in the case of flubendazole would be inhibition of tubulin polymerization. [1] However, clastogens are not considered to have a threshold dose response and since the reduced metabolite of flubendazole shows clastogenic activity, a threshold approach cannot be applied for the positive effects in the *in vivo* micronucleus test. Because of the risk of carcinogenicity linked to the aneugenicity and clastogenicity, no clinical trials in healthy volunteers are allowed and the duration of clinical trials in patients should be limited to single dose only. However, the preclinical efficacy studies showed highest activity upon prolonged exposure after a subcutaneous administration. Such prolonged exposure to a compound with the formation of a potential clastogenic metabolite is associated with a risk for carcinogenicity.

Based on the results of the preclinical toxicity and genotoxicity studies described and discussed in this article, it was concluded that the risk/benefit associated with the use of orally bioavailable flubendazole for the treatment of onchocerciasis in the field is unfavorable. Especially the clastogenicity of the reduced metabolite of flubendazole in combination with the need for exposure beyond one day for efficacy based on the preclinical efficacy studies described by MP Hübner et al. [7] resulted in risk (for carcinogenicity) which outweighed the benefit.

## Methods

### Ethics statement

All work was conducted in accredited laboratories and according to international guidelines.

Safety pharmacology studies in animals were carried out by Charles River Laboratories France Safety Assessment SAS (previously WIL Research Europe-Lyon, France). The study design was in general compliance with the following animal health and welfare guidelines: Guide for the care and use of laboratory animals, 2011; Decree n° 2013–118 relating to the protection of animals used in scientific experiments described in the Journal Officiel de la République Française on 01 February 2013; Directive 2010/63/EU of the European Parliament and of the Council of 22 September 2010 on the protection of animals used for scientific purposes. The Test Facility is fully accredited by the Association for Assessment and Accreditation of Laboratory Animal Care International.

Both rat and dog repeated dose toxicity studies were carried out by Charles River Laboratories Den Bosch B.V. (previously WIL Research Europe B.V., The Netherlands) according to their internal Standard Operating Procedures. The protocols were reviewed and agreed by the Animal Welfare Officer and the Ethical Committee (DEC 14–59) as required by the Dutch Act on Animal Experimentation (February 1997).

The rat in vivo micronucleus study was performed in an AAALAC-approved laboratory of Johnson & Johnson In Belgium. Johnson & Johnson vivarium facilities meet inspection agency standards, and all animals are treated humanely and cared for in accordance with the European [13] and Belgian [14] guidelines, and with the principles of euthanasia as stated in the Report of the American Veterinary Medical Association Panel. [15]

### Test article preparation

Flubendazole has a molecular weight of 313.28, and a molecular formula of C16H12FN3O3.

For the Ames assay, flubendazole was prepared as a solution in dimethyl sulfoxide (DMSO).

For *in vivo* pharmacokinetic studies with intravenous administration, a solution of flubendazole was made in polyethylene glycol 400 (PEG400) with 20% of hydroxypropyl-β-cyclodextrin pH = 4.2 for the rat. For the intravenous pharmacokinetic studies in the jird and the dog, a solution of flubendazole was made in 10 v/v % polyglycol/PEG400 ratio 1–1 and 35 v/v % hydroxypropyl-β-cyclodextrin at pH = 4. For oral pharmacokinetic studies and two-week repeated dose toxicity studies and safety pharmacology studies, the test article/drug product was an amorphous solid dispersion (ASD) powder with a potency of 94.8 mg active ingredient flubendazole per g ASD powder. Because the active ingredient flubendazole only constituted 9.48% of the ASD powder, dose levels were expressed as mg eq./kg body weight meaning mg base/kg body weight. To calculate the amount of ASD powder equivalent to the mg base, conversion factor was as follows: 1 mg base = 10.5 mg drug product. The test article was prepared as a suspension containing 0.5% w/v Methocel A4M (Sigma-Aldrich) in Elix water.

For the rat in vivo micronucleus test, an aqueous solution/suspension in demineralised water containing 10% polyethylene glycol 400, 20% hydroxypropyl-β-cyclodextrin and HCl to pH 1.5± 0.1 was prepared.

### Pharmacokinetic/toxicokinetic studies

The pharmacokinetic studies were performed after single intravenous administration to determine key parameters such as plasma clearance, volume of distribution and half-life, and after single oral administration to determine exposure of flubendazole and its metabolites. The toxicokinetics of flubendazole and its metabolites, which is the determination of the exposure within the toxicology studies were also performed. In both studies, the plasma samples were analyzed individually for flubendazole, H-FBZ and R-FBZ using a qualified LC-MS/MS method. After preparation and addition of the internal standard, samples were precipitated with acetonitrile, mixed and centrifuged. The supernatant was evaporated to dryness under nitrogen flow at 50°C and reconstituted with a mixture of 0.1% formic acid and acetonitrile (90/10, v/v). The extract was injected onto an Acquity UPLC BEH C18 column (50x2.1 mm, 1.7μm particles) (Waters, Milford, USA). The chromatographic system consisted of a Shimadzu SIL30ACMP autosampler and Shimadzu LC30 pumps (Shimadzu, Kyoto, Japan). The mobile phase was a mixture of 1% formic acid and acetonitrile with a flow rate of 0.6 ml/min and a 2.5-minute gradient going from 20 to 60% acetonitrile followed by a 1-minute step gradient to 98% acetonitrile. Mass spectrometric detection was performed on an API4000 triple quadrupole mass spectrometer (Sciex, Framingham, USA) with Turbo Ion Spray ionization operated in positive ion mode. Flubendazole, hydrolyzed flubendazole (H-FBZ) and reduced flubendazole (R-FBZ) were quantified against calibration samples and quality control samples, prepared in the same matrix as the study samples, by means of a qualified analytical method with the lowest limit of quantitation of 0.2, 0.4 and 0.2 ng/ml respectively and an upper limit of quantitation of 3000 ng/ml for all three analytes across the different studies. Flubendazole was administered intravenously by bolus at 1 and 2 mg/kg in male jirds and male rats, respectively or by a short infusion (15 minutes) in male dogs at 0.5 mg/kg. Flubendazole was administered orally by gavage at 20 mg eq./kg in male jirds and male rats and at 35 mg eq./kg in male dogs. The jird and the rat were not fasted. The dogs were fasted overnight. 2h post dose, dogs were given their regular food. After intravenous administration, blood samples were collected at 0.05, 0.25, 0.5, 1, 3, 7 hours after dosing in male jirds (bolus), at 0.0117, 0.333, 1, 2, 4, 7 and 24 hours after dosing in male rats (bolus), at 0.125,0.25 (end of infusion), 0.33, 0.42, 0.50, 0.75, 1.25, 2.25, 4.25, 7.25 and 24.25 hours after the start of infusion in male dogs. After oral administration, blood samples were collected at 0.5, 1, 2, 4, 7 and 24 hours in male jirds, rats and dogs. Plasma concentrations of flubendazole and its 2 metabolites were determined. Several pharmacokinetic parameters were determined: the maximum concentrations (C_max_), the time to reach the C_max_ (t_max_), the half-life (t_1/2_), the area under the curve from time zero to infinity (AUC_0-inf_), the clearance (CL) and the volume of distribution (at steady state Vdss or Vd)

### Safety pharmacology studies

In the safety pharmacology studies, potential undesirable effects of flubendazole on physiological functions in relation to exposure in the therapeutic range and above were investigated. Organ systems evaluated were cardiovascular system, respiratory system (evaluation included in cardiovascular safety study) and central nervous system (CNS).

#### Cardiovascular safety study in dogs [16, 17]

Flubendazole was administered orally via gavage to five telemetered male beagle dogs at single doses of 0, 20, 50 or 120 mg eq./kg, at a dosing volume of 10 mL/kg, on Days 1, 4, 7 and 12, respectively (ascending dosing schedule). Each animal served as its own control, and a minimum wash-out period of 2 days between each dose was respected. Telemetry signals were recorded for at least 1.5 hours before, and 24 hours after dosing. The following parameters were studied: mortality, clinical observations, body weight, arterial blood pressure, heart rate, ECG quantitative parameters and rhythm, respiratory parameters, body temperature, bioanalysis and evaluation of exposure. Blood sampling for exposure was done at a single time-point, 6.5 hours after dosing, time point at which maximum plasma concentration was expected.

#### CNS safety study in rats

Flubendazole was administered orally via gavage to male Sprague-Dawley rats at single doses of 0, 5, 15 or 60 mg eq./kg, in order to evaluate the neurofunctional integrity of the rat (6 male rats/group), using a modified Irwin test procedure [18]. Measurements composing the modified Irwin test are summarized in Table 22. The study was performed in male rats only as is the standard. There was a sex-related difference in plasma exposure for flubendazole in rats but, because there were no indications for sex-related differences in toxicity profile or sensitivity for flubendazole-induced toxicity, there was no reason to deviate from the standard and include also females in this study. The toxicokinetics of flubendazole were studied after completion of the modified Irwin test sessions, by treating the 6 rats previously treated with the vehicle with the dose of 60 mg eq./kg. Sampling for plasma exposure was performed on a separate day to not interfere with the neurofunctional observations. Sampling points were pre-dose, 30 minutes, 1, 2, 4, 7 and 24 hours after dosing. Maximum mean plasma concentrations (C_max_), their times of occurrence (T_max_), the time of the last quantifiable mean concentration (t_last_) and the mean plasma concentrations at 24 hours post-dose (C_24h_) were the observed values. Areas under the mean plasma concentration time curves (AUC) were calculated. C_max_, T_max_ and AUC_0-24h_ are described in this paper. Mortality, behavioural observations in the cage, during handling and while in an open field, general clinical observations, body weight and rectal temperature were evaluated on Day 1 (day of single administration), Day 2 and Day 7.

**Table 22:** Modified irwin test parameters.

Parameter observed	Measurement
Spontaneous activity—Central Nervous System excitability	
Home cage posture	R
Tremors	R and D
Convulsions	R
Stereotypies	R and D
Vocalisations	Q
Ease of removal	R
Handling reactivity	R
Body tone	R
Fur appearance	R
Rearing (supported and non-supported)	C
Grooming	C
Arousal	R
Autonomic functions	
Palpebral closure	R
Exophthalmus	Q
Lacrimation	R
Crusts around eyes	Q
Salivation	R
Piloerection	Q
Defecation	C
Urination	C
Gait and muscle tone	
Gait	R and D
Righting reflex	R
Grip strength	Co
Motor and sensory reflexes	
Approach response	R
Touch response	R
Glove snap response	R
Tail pinch response	R
Catalepsy	R
Physiological parameters	
Rectal temperature	Co

R: rank order data, Q: quantal data, C: count data, D: descriptive data, Co: continuous data

Safety pharmacology studies in animals were carried out by Charles River Laboratories France Safety Assessment SAS (previously WIL Research Europe-Lyon, France), following internal Standard Operating Procedures and applicable ICH guidelines (ICH S7A, S7B) and were GLP regulated [19, 20, 21, 22].

### Repeated dose toxicity studies and toxicokinetics [23, 24]

#### Two-week repeated dose toxicity study in the rat

Flubendazole was administered by oral gavage for 14 or 15 consecutive days to Sprague Dawley rats. Dose levels for males were 0, 5, 15 and 30 mg eq./kg/day; dose levels for females were 0, 2.5, 5 and 10 mg eq./kg/day. Dose levels in females were lower compared to males because exposure in females was at least twice the exposure in males for flubendazole after a similar single dose. Because of the higher exposure, more pronounced toxicity findings were observed in females when compared with males. A similar group of rats received the vehicle only, according to the same regimen and served as a vehicle control group. Initially, the study included a recovery period of one month to investigate the reversibility of any possible toxicological effect induced by repeated oral administration of flubendazole after treatment was stopped. For this purpose, extra rats for the vehicle and the high dose group (recovery animals) were added to the main toxicity study (main study: 10 rats/sex/group, for recovery: 5 rats/sex/group added to the vehicle and high dose groups only). However, all high dose animals including animals assigned for recovery, were killed in extremis on Day 7, 8 or 11 of the dosing period. Since there were no high dosed animals left to evaluate recovery after treatment was stopped, recovery animals of the control group were necropsied on Day 4 of Recovery. In this study, the following parameters were studied: mortality, clinical and ophthalmoscopic observations, body weight and weight gain, food consumption, haematology, coagulation, clinical biochemistry, urinalysis, organ weights, gross pathology and histopathology. The toxicokinetic parameters for flubendazole and the 2 metabolites, reduced flubendazole and hydrolyzed flubendazole were also determined in satellite animals (3 rats/sex/group added to the vehicle-dosed group, and 6 rats/sex/group added to the flubendazole-dosed groups as satellite animals to sample for toxicokinetic analysis). The toxicokinetic parameters determined were maximum mean plasma concentrations (C_max_), their times of occurrence (T_max_), the time of the last quantifiable mean concentration (t_last_) and the mean plasma concentrations at 24 hours post dose (C_24h_) as the observed values. Areas under the mean plasma concentration-time curves (AUC) were calculated. C_max_ and AUC_0-24h_ are described in this paper. Samples for toxicokinetic analysis were taken on day 1 and day 14 of the dosing period from satellite animals for all flubendazole-dosed groups except the high dose groups and on day 1 and day 10 of the dosing period for the high dose groups. In the vehicle dose groups, samples were taken on day 3 and day 11 of the dosing period. Sampling time points were 30 minutes, 1, 2, 4, 7 and 24 hours after dosing for the flubendazole-dosed groups and 1 and 7 hours after dosing for the vehicle dosed groups. Animals of vehicle-dosed groups were sampled to confirm absence of exposure to flubendazole in these animals.

#### Two-week repeated dose toxicity study in the dog

Flubendazole was administered orally via gavage to male and female Beagle dogs (3/sex/group) at dosages of 0, 20, 40 or 100 mg eq./kg /day during 14 or 15 consecutive days. The high dose group received twice daily 50 mg eq./kg, with a 6-hour interval. A similar group of dogs received the vehicle only (once daily) and served as a vehicle control group. In addition, a recovery period of 1 month was added to investigate the reversibility of any possible toxic effect induced by repeated oral administration of flubendazole. For this purpose, 2 extra dogs/sex for the vehicle, mid and high dose group were added to the study.

The following parameters were studied: mortality, clinical and ophthalmoscopic observations, ECG and heart rate, body weight and weight gain, food consumption, haematology, coagulation, clinical biochemistry, urinalysis, organ weights, gross pathology and histopathology. The toxicokinetic parameters for flubendazole and 2 metabolites, reduced flubendazole and hydrolyzed flubendazole were also determined. Maximum plasma concentrations (C_max_), maximum plasma concentrations after the first daily dose (C_max1_) and after the second daily dose (C_max2_), their times of occurrence (T_max_, T_max1_ and T_max2_ respectively), the time of the last quantifiable concentration (t_last_) and the plasma concentrations at 24 hours post dose (C_24h_) were the observed values. Areas under the plasma concentration-time curves were calculated. C_max_ and AUC_0-24h_ are described in this paper. Samples for toxicokinetic analysis were taken from all flubendazole-dosed dogs on day 1 and day 14 of the dosing period and from vehicle dosed dogs on day 3 and day 13. Sampling time points were pre-dose, 30 minutes, 1, 2, 4, 7 and 24 hours after dosing in the vehicle, low and medium dose groups. In the high dose groups, dogs were sampled 30 minutes, 1, 2, 4, 6 (before second dose), 7, 9 and 24 hours after the first dose. Animals of vehicle-dosed groups were sampled to confirm absence of exposure to flubendazole in these animals.

Both rat and dog studies were carried out by Charles River Laboratories Den Bosch B.V. (previously WIL Research Europe B.V., The Netherlands) according to their internal Standard Operating Procedures. They were GLP regulated [25] and were conducted in compliance with the ICH regulatory guidelines ICH M3 [23].

### Genotoxicity assessments [26]

Genotoxicity assessments were performed to detect if flubendazole induces genetic damage.

#### Ames test

Flubendazole was tested to evaluate its mutagenic potential by measuring its ability to induce reverse mutations at selected loci of several strains of Salmonella typhimurium and at the tryptophan locus of Escherichia coli strain WP2 uvrA in the presence and absence of an exogenous metabolic activation system. The tester strains used were the Salmonella typhimurium histidine auxotrophs TA98, TA100, TA1535 and TA1537 as described by Ames et al. [27] and Escherichia coli WP2 uvrA as described by Green and Muriel [28]. DMSO was used as the vehicle. Aroclor 1254-induced rat liver S9 was used as the metabolic activation system. The S9 was prepared from male Sprague-Dawley rats that were injected intraperitoneally with Aroclor 1254 (200 mg/mL in corn oil) at a dose of 500 mg/kg body weight, five days before sacrifice. The test system was exposed to the test article via the plate incorporation methodology originally described by Ames et al. [27] and updated by Maron and Ames [29]. The dose levels tested were 10.0, 25.0, 50.0, 75.0, 150, 300, 600, 1200 and 2000 μg per plate. The top dose was limited to 2000 μg per plate based on solubility but was still compliant with OECD [30] based on the observation of precipitate on the assay plates. During dosing, precipitate was observed beginning at 300 μg per plate. During scoring and background lawn evaluation, no toxicity was observed, and precipitate was observed beginning at 600 μg per plate.

The Ames test was performed by BioReliance Corporation, Rockville, MD 20850, United States of America, according to their internal Standard Operating Procedures and was GLP regulated [31].

#### *In vivo* micronucleus test in rats

Flubendazole was administered orally via gavage to young male and female rats to assess its potential to induce structural and/or numerical chromosome aberrations in bone marrow and peripheral blood immature erythrocytes. Male and female rats (5 rats/sex/group) were dosed once daily with flubendazole at 0.15, 0.5, 1.5, 5, 15, 50 and 150 mg/kg body weight/day for two consecutive days prior to the collection of bone marrow and peripheral blood at 24 hours after the last administration. A similar group of rats (5 rats/sex/group) received the vehicle only, according to the same regimen and served as a vehicle control group.

The number of micronucleated and nonmicronucleated reticulocytes (RETs), and the number of micronucleated and nonmicronucleated normochromatic erythrocytes (NCEs) in peripheral blood was recorded by the flow cytometer. Data acquisition was stopped when 20,000 RETs had been collected. The percentage of RETs was calculated (number of reticulocytes over total number of mature and immature erythrocytes) as an index for bone marrow toxicity. The percentage of micronucleated RETs (number of micronucleated reticulocytes over total number of reticulocytes) was calculated as a measure for induction of structural and/or numerical chromosome aberrations by the test item.

Bone marrow smears were evaluated by fluorescence microscopy. Analysis of red blood cell proliferation was based on the proportion of polychromatic erythrocytes assessed by counting a total of 1000 erythrocytes (polychromatic plus normochromatic erythrocytes; PCEs + NCEs) per rat. Analysis of red blood cells for micronuclei was based on a total of 2000 PCEs per rat and the number of micronucleated PCEs. At the same time, the number of micronucleated NCEs was also recorded in the fields containing these 2000 PCEs.

The toxicokinetic parameters for flubendazole and the 2 metabolites, reduced flubendazole and hydrolyzed flubendazole were also determined in satellite animals (3 rats/sex/group added to the flubendazole-dosed groups as satellite animals to sample for toxicokinetic analysis). The toxicokinetic parameters determined were peak plasma concentrations (C_max_) and corresponding peak times (T_max_). The area under the plasma concentration time curve (AUC) were calculated. C_max_ and AUC_0-24h_ are described in this paper. Samples for toxicokinetic analysis were taken on day 1 of dosing from the satellite animals. Sampling time points were 30 minutes, 1, 2, 4, 7 and 24 hours after dosing.

This study was classified as a non-GLP study. This study was carried out based on the current OECD guideline 474 (1997) [32] The study was performed in an AAALAC-approved laboratory of Johnson & Johnson.

### Statistical analysis

#### 2-week repeated dose toxicity studies

In the rat study, the following statistical methods were used to analyze the data: If the variables could be assumed to follow a normal distribution, the Dunnett test (many-to-one t-test) based on a pooled variance estimate was applied for the comparison of the treated groups and the vehicle control groups for each sex. The Steel-test (many-to-one rank test) was applied instead of the Dunnett-test if the data could not be assumed to follow a normal distribution. The Fisher-exact test was applied to frequency data. All tests were two-sided and in all cases p < 0.05 was accepted as the lowest level of significance.

In the dog study, no statistical analysis took place due to the limited number of animals in the study.

#### Genotoxicity assessments

In the *in vivo* micronucleus test, the significance of differences between each test item group (and positive control group) and the vehicle control group was assessed by the following statistical methods: For mortality and clinical observations, Fisher Exact Probability test was applied. For body weight/weight gain and bone marrow data of the micronucleus test, the Mann-Whitney U test was applied. For flow cytometric analysis data, data (the percentage of RETs and the percentage of micronucleated RETs), were checked for non-normality by applying the Shapiro-Wilk test. When data were not normally distributed, the percentage of RETs was square root transformed and the percentage of micronucleated RETs was log transformed and subsequently rechecked for non-normality using the Shapiro-Wilk test. When data were normally distributed, homogeneity of variances was assessed through the Levene’s test. When data were either not normally distributed or homogeneity of variances could be rejected, then Dunn’s test (with a closed multiple comparison procedure following Holm) was applied for comparison of the test article groups to the vehicle control group. Otherwise, Dunnett’s test was applied. The above procedure was adopted for the comparison of all dose groups versus vehicle control and for the comparison of vehicle control versus the positive control. In the last comparison, where only two groups are compared, Dunnett’s test reduces to a standard t-test and Dunn’s test reduces to a Wilcoxon-Mann-Whitney test. In addition, Jonckheere Terpstra trend tests for dose trends were performed. All analyses of flow cytometric analysis date were performed with SAS version 9.2. All tests were performed at a significance level of 5% or less.
